# Mobile Exergaming for Health—Effects of a serious game application for smartphones on physical activity and exercise adherence in type 2 diabetes mellitus—study protocol for a randomized controlled trial

**DOI:** 10.1186/s13063-017-1853-3

**Published:** 2017-03-06

**Authors:** Christoph Höchsmann, Steffen P. Walz, Juliane Schäfer, Jussi Holopainen, Henner Hanssen, Arno Schmidt-Trucksäss

**Affiliations:** 1 Division of Sports and Exercise Medicine, Department of Sport, Exercise and Health, University of Basel, Birsstrasse 320 B, 4052 Basel, Switzerland; 2 Swinburne University, Melbourne, Australia; 3 RMIT Europe, Barcelona, Spain

**Keywords:** Type 2 diabetes, Physical activity, Exergaming, Exercise adherence, Diabetes management

## Abstract

**Background:**

Exergaming is a novel approach to increase motivation for regular physical activity (PA) among sedentary individuals such as patients with type 2 diabetes mellitus (T2DM). Because existing exergames do not offer fitness-level adjusted, individualized workouts and are normally stationary (TV bound), thus not enabling PA anywhere and at any time, we developed a smartphone-based, game-like software application (MOBIGAME) specifically designed for middle-aged T2DM patients to induce a healthier, more active lifestyle as part of successful T2DM treatment and management. In a randomized controlled trial we aim to examine whether our smartphone-based game application can lead to increases in daily PA in T2DM patients that are persistent in the mid to long term and whether these increases are greater than those in a control group.

**Methods:**

This study is designed as a randomized controlled trial. We plan to recruit a total of 42 T2DM patients [45-70 years, body mass index (BMI) ≥25 kg/m^2^, low daily PA, regular smartphone use].

The experimental intervention (duration 24 weeks) includes individualized multidimensional home-based exercise and daily PA promotion administered through MOBIGAME. The control intervention consists of a one-time standard lifestyle counseling including the promotion of baseline activities.

The primary outcome is daily PA measured as steps per day. Secondary outcome is exercise adherence measured via the usage data from the participants’ smartphones (experimental intervention) and as self-recorded exercise log entries (control intervention).

We will test the hypothesis that there will be differences between the experimental and control group with respect to post-interventional daily PA (as well as all other outcomes) using analysis of covariance. For each analysis, an estimate (with 95% confidence interval) of the difference in outcome between both groups will be reported.

**Discussion:**

This research will investigate the effectiveness of a novel smartphone-based, game-like software application to be used as a way to promote regular daily PA among inactive T2DM patients. The results of this trial may have important implications for future PA-promoting interventions and provide relevant information for the general transferability of such applications to be used as part of the treatment in other chronic diseases.

**Trial registration:**

ClinicalTrials.gov, NCT02657018. Registered on 11 January 2016. Last status update on 3 May 2016. Kofam.ch, SNCTP-number:SNCTP000001652. Registered on 21 January 2016.

**Electronic supplementary material:**

The online version of this article (doi:10.1186/s13063-017-1853-3) contains supplementary material, which is available to authorized users.

## Background

### Overweight and Type 2 Diabetes mellitus—defining the target group

Overweight and obesity have become a global public health issue over the past decades reaching worldwide prevalences of 39% (overweight) and 13% (obesity) in 2014 and thereby having more than doubled since 1980 [1]. Along with these increases, type 2 diabetes mellitus (T2DM), a common comorbidity of overweight and obesity, has also seen dramatic increases in prevalence. In 2012, 9.3% of the US population was diagnosed with T2DM and 27.5% with prediabetes, the precursor stage to T2DM [2]. The direct and indirect medical costs accrued by patients with T2DM in the US in 2012 accounted for an estimated $245 billion, while people diagnosed with diabetes created 2.3 times higher average medical expenditures than people without diabetes, underlining the enormous financial burden that is caused by this disease.

Both obesity and T2DM are associated with a high level of inactivity [3, 4] that aggravates the physical and mental health status and constitutes a crucial factor in the development of several comorbidities such as hypertension, dyslipidemia, cardiovascular disease, and kidney and nerve disease [2]. In 2008, 9% of all premature deaths worldwide were attributed to physical inactivity, making its impact comparable to that of smoking, an established risk factor [5]. Physical inactivity is furthermore responsible for 7% of the burden of disease from T2DM [5], and it has been shown that decreased maximum oxygen consumption (VO_2max_) is among the earliest indicators of insulin resistance and T2DM [6], thus representing an important risk factor for disease progression. It has further been shown that participation in regular physical activity (PA) improves blood glucose control [7, 8] and can prevent or delay onset of T2DM [9–12] and its comorbidities [13, 14]. Individuals who engage in regular PA, such as the American College of Sports Medicine (ACSM) recommended 30 min of brisk walking on 5 days per week, have a 30% lower risk of developing T2DM as compared to sedentary individuals [13, 15]. In individuals with T2DM, increased glucose uptake into active muscles during exercise as well as acute improvements in systemic insulin action lasting from 2 to 72 h post exercise have been reported [13]. Chronic effects of both aerobic and resistance training include improvements in insulin action, blood glucose control and fat oxidation [13].

In addition to the T2DM-induced impairments in the individual’s glucose metabolism, severe decreases of skeletal muscle mass have been reported in older adults with T2DM, demonstrating the negative effects of the disease on body composition [16–18]. These losses of muscle mass are most profound in the extremities and especially in the lower limbs, while they are independent of body weight change over time [16]. In general, the loss of muscle mass is paralleled by significant decreases in muscle strength [19]. However, a 50% more rapid decline in the knee extensor strength was found in older adults with T2DM compared to non-diabetics of the same age even after controlling for losses of leg muscle mass [19]. This indicates a more rapid decline in muscle quality, suggesting that T2DM may result in impairments in muscular function of the lower extremities as well, not necessarily accompanied by a loss of muscle mass [19]. In addition to reduced levels of muscle strength, patients with T2DM often show reduced levels of aerobic exercise capacity due to an impaired recruitment of skeletal muscle capillaries [20] and an impaired bioenergetics capacity of skeletal muscle mitochondria [21]. In patients with poor disease management and existing microvascular complications, these impairments are even further increased and significantly add to the cardiovascular risk [22]. Factors such as reduced muscle strength, decreased aerobic capacity and rapid accumulation of lactate during exercise as a result of the impaired muscle energetics might also be responsible for early muscle exhaustion, lower exercise tolerance and fatigue in T2DM [23]. Especially peripheral fatigue of the skeletal muscles, which arises from a combination of neurological, musculoskeletal and metabolic changes, such as reductions in glycogen stores, reduced oxygen consumption during activity and changes in muscle fibers induced by physical inactivity and aging, are of great importance in T2DM [23]. Fatigue has far-reaching and serious consequences for patients with T2DM as it interferes with self-reported quality of life [24] and diabetes self-management [23, 24]. Fatigue further leads to greater physical inactivity [25] and thus further facilitates muscle atrophy and declines in muscle strength especially in the lower limbs [16–20]. Reduced muscle quality and lower aerobic capacity subsequently amplify fatigue with all of its physiological and psychological ramifications [23], ultimately resulting in a vicious cycle that needs to be broken since the effects of regular PA in T2DM are very promising [7–15], especially since increases in muscle mass of the lower limbs are crucial for improvements in insulin sensitivity and thus successful T2DM management [26].

### Exergaming as a motivating way to increase exercise adherence—reaching the target group

For any PA-promoting lifestyle intervention to be effective, motivation to start and adhere to the lifestyle changes is crucial. As explained before, T2DM patients usually show very low levels of PA [4]. Lack of motivation, missing social support and disease-related implications such as fatigue or pain are the main reasons why patients with T2DM cease to participate in PA promoting programs [27]. Exergames, the coupling of PA and video gaming, offer an enjoyable and promising new approach to increase PA in individuals that are among the least likely to engage in regular PA [28, 29]. It has been shown that these games can enhance health-related learning and even behavior change [28] because they are experiential and interactive and immerse the player in worlds that offer compelling challenges and immediate progress feedback, ultimately leading to improved diabetes self-management and better health outcomes [28, 30]. The Nintendo Wii Fit™ Plus exergame has been shown to motivate elderly patients with T2DM to engage in increased voluntary and regular PA and consequently improve long-term blood glucose control (HbA1c), body composition and quality of life [31]. It has also been demonstrated that exergames such as “Dance Dance Revolution” meet vigorous PA requirements for improving or maintaining physical fitness in a wide range of adults [32]. In a previous study with T2DM patients [33], we were able to show that selected Nintendo Wii Fit™ Plus exercises (Boxing, Obstacle Course and Cycle Island, played for bouts of 10 min each) bear the potential to produce exercise intensities that correspond to moderate-intensity PA [67-70% of the maximum heart rate (HR_max_) and a mean oxygen consumption (VO_2mean_) of 40% of the peak oxygen consumption (VO_2peak_) previously established in an all-out treadmill test]. A moderate to strong correlation between treadmill VO_2peak_ and both exergame VO_2peak_ and exergame VO_2mean_ in the same study indicated that subjects with a higher fitness level were able to exercise at a higher intensity during exergame play than individuals with a lower fitness level. These results suggest that carefully selected Wii Fit™ Plus game modes can potentially induce health benefits similar to common aerobic exercise and would thus be able to help improve the glucose metabolism and reduce the risk of premature all-cause as well as cardiorespiratory mortality in T2DM patients.

In a systematic review [34] it has further been shown, however, that the current state of research in this field contains significant research gaps. The true effectiveness of the currently available exergames in regard to changes in objectively measured intensity parameters of PA [i.e., VO_2,_ energy expenditure, metabolic equivalent (MET), heart rate or activity counts] remained unclear as a result of the vastly differing study designs and greatly varying exergame expositions used in the respective studies. It was further noted that future exergame interventions will need to be conducted in a home-based setting rather than in the laboratory to fit into and be part of one’s daily routine. This is necessary to truly examine the suitability of exergames to promote daily PA. In addition it was pointed out that future exergames would only be able to contribute to a healthier, more active lifestyle if they keep players motivated in the long term. This lasting motivation is especially important among a target group that is likely to be sedentary, such as that of T2DM patients who usually do not show a lot of intrinsic motivation to be physically active [34]. At the same time, future exergames, unlike the currently available exergames, need to feature game modes with a minimum duration of 10 min, as suggested by current guidelines [35], and an exercise intensity that corresponds to moderate PA [35] to be able to offer effective workout alternatives for the target group of T2DM patients [34]. To meet the different fitness levels of the different players, future exergame designs should further offer more tailored intensity levels and a more individualized intensity progression than the currently available exergames do.

### Development of the MOBIGAME application

To address the shortcomings of existing exergames we—a group of researchers at different universities, from the fields of sports medicine and (serious) game as well as behavioral design, in collaboration with a long-standing, commercial serious game development studio—developed a mobile smartphone-based, game-like software application and platform (MOBIGAME) specifically designed for the needs of T2DM patients and those at risk of developing T2DM, with the goal to induce a healthier, more active lifestyle in the target group.

The use of smartphones and mobile apps especially among middle and older age groups has grown exponentially in the past years [36] with 50% of casual gamers being between 35-64 years [37]. These age groups are also disproportionately impacted by chronic diseases; 60% of adults aged 50-64 years have at least one chronic condition [37] such as obesity and T2DM [2]. Using these technologies to provide tools for diabetes prevention and management is timely and offers a cost-effective opportunity to directly reach a larger segment of the target population than traditional diabetes prevention programs. At the same time, smartphones’ integrated sensors and actuators as well as wearable sensor devices enable personalized diabetes prevention and can thereby significantly improve sustainability and dissemination of such intervention programs [38].

It has been shown [38] that the replacement of the group sessions in a diabetes prevention intervention by a mobile app in combination with in-person sessions, focusing on lifestyle-enhancing personal skills, is able to induce increases in daily PA of up to 3100 steps per day in an 8-week period compared with baseline. Even after 20 weeks daily PA remained elevated at approximately 2500 steps per day over baseline. It is noteworthy, however, that after the peak in daily PA at week 8, mean daily steps continuously decreased until the end of the intervention while this decline was paralleled by a likewise continuous decrease in adherence to the mobile app [38]. Adherence decreased almost linearly from 85% at week 1 to 40% at week 20. There is good reason to believe that this decline in adherence influenced the amount of daily PA negatively. In fact, it is possible that adherence levels would have been higher and more persistent in the study population of T2DM patients if a more game-like and thus more enjoyable approach had been used.

The effectiveness of a smartphone-based gaming application in increasing players’ daily PA is currently demonstrated by the enormous success of Pokémon GO [39], which attracted over 65 million users within the first week of its launch [40]. While this app undeniably leads to an at least GPS-tracked increase in daily PA (since steps are not measured, other ways to search for Pokémon, such as driving, cannot be ruled out), it is missing fundamental components to make it a safe exercise tool. First, some public spaces designated for Pokémon use are simply inappropriate or even dangerous. This has led to several accidents while playing Pokémon GO due to inattention to surroundings while walking or even driving [40, 41], presenting serious risks especially to the more vulnerable, often easily distracted pediatric population [42]. Second, the app does not take the player’s cardiorespiratory fitness or possible health restrictions into account when placing Pokémon in the surrounding environment. Especially for low-fitness players with chronic diseases, such as T2DM, the distances that need to be covered in order to find Pokémon may cause an unhealthy physical overexertion in the short-term and lead to overuse injuries in the long term.

Therefore, for it to be a safe exercise tool especially for individuals with chronic diseases, an interactive smartphone game needs to feature a structured, guideline-concordant and fitness-adjusted exercise intervention that uses a variety of the device’s sensors to track and further tailor the player’s PA. While safely promoting regular physical activity as part of a lifestyle change, this approach could help further reduce staff expenses compared to interventions using in-person sessions [38] or cognitive-behavioral telephone support sessions led by a psychologist [43] while still ensuring an effective diabetes prevention and long-term treatment.

The MOBIGAME project sought to implement evidence-based sports scientific knowledge into the production of an entertaining and interactive mobile software application that offers individualized and structured exercise regimens and that can be played at home as well as on the go. Special regard was paid to exercises that can be tracked via the mobile phone’s sensors (camera, accelerometer/pedometer, position sensor, noise recognition or Global Positioning System, GPS) to design a clearly exercise-oriented game that is challenging and also responds to an individual player while including compliance, monitoring and motivational aspects. A key component of the application is the integration of exercise tests such as the 1-min Sit-to-Stand Test (STS) [44] and the 6-Minute Walk Test (6MWT) [45] to assess the exercise-related capabilities of each individual user. In the application, these tests are used as baseline measurements to assess the user’s fitness level in a home-based setting and thereby make it possible to build tailored exercise programs for each individual user, even those with severely compromised fitness. Exercise regimens integrated into the game application include strength and endurance workouts as well as balance and flexibility exercises and follow the ACSM and European Association for Cardiovascular Prevention & Rehabilitation (EACPR) principles of exercise training [46, 47]. Additionally, daily PA is promoted and tracked via the user’s smartphone sensors. Personalized daily (and weekly) step goals are set for each user. The suitability of the developed strength exercises including the different intensity/difficulty levels has been evaluated in a qualitative user study (*N* = 12) with the target group (unpublished data). In this user study, first the baseline tests were conducted, and the individual fitness levels were assessed using population-based reference values [44]. Based on the results of the baseline tests, and hence the fitness assessment of the participant, exercises and respective difficulty levels were selected from a pool of upper-limb, lower-limb, core and back exercises. The main objective of the user study from the sports medical point of view was to validate the categorization of the different difficulty levels and to evaluate whether the exercises that were selected based on the results of the STS were in fact feasible for each participant and thus concurrent with our fitness assessment. From the game and behavioral design's, as well as from the game developers’ point of view, the main objective of the user study was to assess the playability of the game concept and the appeal to the target group.

The motivational approach chosen for MOBIGAME to increase PA while obeying the abovementioned principles follows the promotion of the players’ self-efficacy and specifically their personal mastery [48–50]. Rather than directly targeting a particular behavior (i.e., increased PA), successful exergames target mediators (i.e., self-efficacy), which, in turn, affect the behavior and then the health outcome of interest [51]. In this context, self-determination, namely (1) perceived competence (the player’s actions are responsible for the success in the game), (2) perceived autonomy (the player feels in charge of the own choices in the game) and (3) relatedness (a sense of belonging or connection), has repeatedly shown a strong relationship with the experience of enjoyment as well as sustained engagement and lasting behavior change in multiple domains [52, 53]. The enjoyment arising in the context of the game experience supports and facilitates the behavior change as it creates a positive emotional state of playful enjoyment in which the player is intrinsically motivated, meaning that the value of the experience is the experience itself, rather than the experience being instrumental in achieving something else [52].

Through the use of MOBIGAME, the players will further learn that their regular PA is directly responsible for the achievements in the game and thus has an immediate and noticeable impact [54]. The gameplay design thus represents a kind of “reflection-on-action” approach [55] in which the players’ motivation leads to adjustments in real-life behaviors (i.e., walking more steps per day) in response to events or incidents in the game’s story line.

As MOBIGAME uses sensor tracking to verify the execution and completion of workouts that requires a specific, sensor-dependent (camera, accelerometer or position sensor) position of the device for each exercise, an actual gamification of the exercises such as a visualization of the execution on the phone’s screen is not feasible and the adaption of the proposed movement-based game guidelines [56] not always possible. Since the screen of the average smartphone is rather small compared to a TV, however, gamification of the exercises as used in traditional exergames such as Nintendo Wii or Xbox Kinect would also not present an acceptable option for use by our target group. Therefore, the game guidelines [56] were taken into account during the development of the game design, but adapted to suit the idea of our mobile game application. Embedding structured and individualized strength and endurance workouts as well as promotion of daily PA into the story line of a mobile phone-based game, which uses self-efficacy as a key motivational aspect by giving additional meaning to the player’s PA, offers a promising and feasible solution to increase PA and to be effective in the target group in the long term.

### Main hypothesis

The experimental intervention (use of MOBIGAME) is more effective for increasing daily PA measured as steps per day than a control intervention after 24 weeks.

## Methods

### Study overview: target group, setting, design and registration

**Fig. 1:**
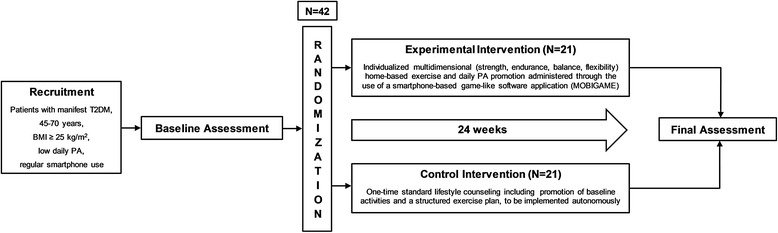
Study flow chart. T2DM, type 2 diabetes mellitus; BMI, body mass index; PA, physical activity

The study targets patients with non-insulin-dependent diabetes mellitus, 45-70 years of age, with a body mass index (BMI) ≥25 kg/m^2^, who use a smartphone regularly and show low and irregular daily PA. The study aims to include a total of 42 German-speaking patients in the Basel (Switzerland) metropolitan area. The setting of the intervention is the patients’ everyday environment (home, work, places of leisure time activities), where the MOBIGAME application is used and the included exercises are carried out. The study is a prospective randomized controlled trial and has been registered with the US National Institute of Health’s Protocol Registration and Results System (PRS) on https://clinicaltrials.gov/(NCT02657018) as well as with the Federal Office of Public Health’s (FOPH) portal for human research in Switzerland on http://www.kofam.ch/(SNCTP-number:SNCTP000001652). For a study flow diagram, see Fig. 1. The SPIRIT checklist is provided as Additional file 1. Scientific lead, study management and coordination, patient information and recruitment in cooperation with the Clinic of Endocrinology, Diabetes and Metabolism of the University Hospital Basel, measurements and statistical analyses are performed by the Department of Sport, Exercise and Health of the University of Basel.

### Eligibility criteria

This study aims at tertiary prevention and therefore patients have to be diagnosed with a manifest non-insulin-dependent diabetes mellitus (doctor diagnosed) to be eligible to participate. They further have to be overweight (BMI ≥25 kg/m^2^), between 45 and 70 years of age, have used a smartphone regularly during the last year before the study and give their written informed consent. Because questionnaires will be filled out as part of this study, sufficient proficiency of German is required. Patients will be excluded if any one of the following criteria is applicable:

- ▪ Impaired physical mobility (participants have to at least be able to walk short distances indoors without a walking aid and without help of another person).
- ▪ Regular PA before the study (≥150 min moderate intensity daily PA per week or >1 endurance or strength training session per week of more than 30 min in duration).
- ▪ Other clinically significant concomitant disease states (e.g., renal failure, hepatic dysfunction, cardiovascular disease, etc.).
- ▪ Inability to follow the procedures of the study, e.g., due to language problems, psychological disorders, dementia, etc., of the participant.
- ▪ Resting systolic blood pressure >170 mmHg, resting diastolic blood pressure >100 mmHg.
- ▪ Participation in other clinical studies in the last 4 weeks.

### Experimental and control intervention

#### Overview

The duration of the intervention is 24 weeks. In the experimental group the intervention consists of:

1. Individualized multidimensional (strength, endurance, balance, flexibility) home-based exercise and daily PA promotion administered through the use of MOBIGAME following established PA guidelines [47].
2. Consultations provided by a sports medical expert via telephone, including personal attention and instruction as well as technical support

In the control group the intervention consists of:

1. One-time lifestyle counseling (standard of care) including the promotion of baseline activities of daily life [57] as well as a structured exercise plan including strength and endurance exercises with moderately increasing intensity and duration, essentially comparable to the content of MOBIGAME, which is to be implemented autonomously.
2. Consultations provided by a sports medical expert via telephone, including personal attention and instruction.

#### Common aspects for both groups

**Fig. 2:**
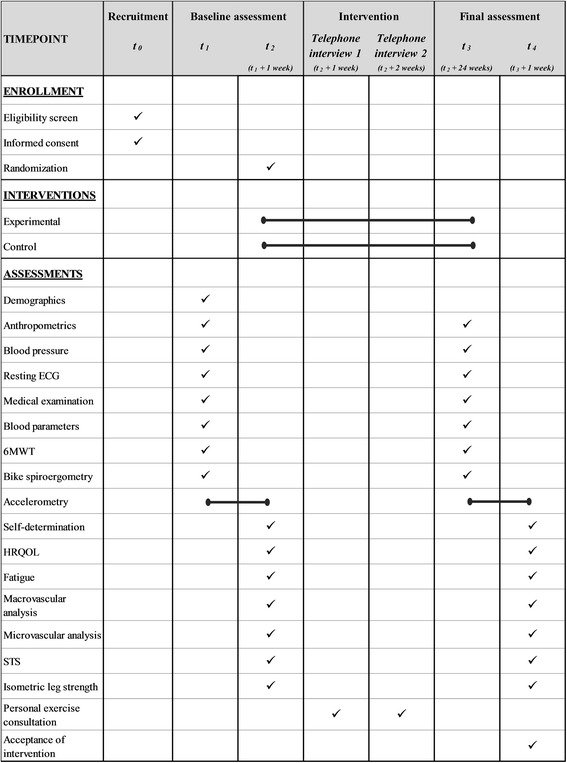
Schedule of enrollment, interventions and assessments according to the Standard Protocol Items: Recommendations for Interventional Trials (SPIRIT). 6MWT, 6-Minute Walk Test; ECG, electrocardiogram; HRQOL, health-related quality of life; STS, Sit-to-Stand Test

In both groups a sports medical expert will provide a given number of personal exercise consultations on the telephone (weeks 1 and 2) as well as a face-to-face consultation at week 0, thus guaranteeing that the control group receives the same number of consultations as the experimental intervention group. For an overview of the study schedule, see the Standard Protocol Items: Recommendations for Interventional Trials (SPIRIT) schedule [58] (Fig. 2).

#### Experimental intervention details

The experimental intervention aims at improving participants’ motivation to exercise in the short and especially in the mid to long run through the use of MOBIGAME and thereby increasing daily PA. MOBIGAME features numerous different exercises (see Table 1) and exercise regimens including strength and endurance workouts as well as balance and flexibility exercises (a total of over 130 exercises) that concur with generally accepted PA guidelines [46, 47]. The strength exercises span over a wide range of different difficulty and intensity levels. Each exercise contains up to 15 variants with up to 30 different levels regarding repetitions, bouts and duration for each variant. All exercises are designed in a way that makes it possible for players with any fitness level to gradually progress with their workout routine while increasing workout intensities at an individualized pace.

**Table 1:** Overview of exercises included in MOBIGAME

Category	No.	Exercise (no. of variants)
Strength	1	Hip/knee extension, dynamic (15)
	2	Knee extension, static (3)
	3	Leg lift, frontal/lateral (9)
	4	Arm elevation (7)
	5	Arm abduction (7)
	6	Elbow extension/shoulder protraction (6)
	7	Back extension, dynamic (6)
	8	Upper back/shoulder extension, static (4)
	9	Abdominals, static (5)
	10	Abdominals, dynamic (10)
Endurance		**Aerobics**
	1	March (2)
	2	Tap (6)
	3	Step touch (4)
	4	Out-In (2)
	5	V-step (2)
	6	Box-step (2)
	7	Grapevine (2)
	8	Leg curl (2)
	9	Knee lift (2)
	10	Front kick (2)
	11	Heel dig (2)
		**Walking**
	1	Continuous (1)
	2	Interval (4)
Flexibility	1	Chest/upper back (2)
	2	Torso (1)
	3	Neck (2)
	4	Lateral torso (1)
	5	Ischiocrural muscles (2)
Balance	1	Two-leg stand (5)
	2	Tandem stand (5)
	3	One-leg stand (5)

Data captured in bold indicates that all Aerobics exercises as well as the two Walking exercises are sub-categories of the Endurance exercises

Endurance workouts include a wide variety of walking exercises with different walking speeds and exercise modalities as well as aerobic workouts (Table 1). Walking exercises are to be conducted outdoors and include continuous trainings as well as interval trainings. Since the 6MWT speed is used as a reference, the proposed walking speeds during the workouts are individually adjustable (e.g., 70% of 6MWT speed). To prevent players from being inattentive to their surroundings because of smartphone distraction, walking exercises are not visualized on the smartphone’s screen. Instead, players are advised to keep the phone in their pocket throughout the exercise, which in addition will guarantee a more accurate tracking of the players’ step count during the exercise. Before the start of each walking exercise, along with the exercise instruction, the players are further advised to ensure a safe environment that is suitable for the upcoming walking exercise. This precaution in design is based on prior research on location-based games that involve explorative, walking activities in public space; it had been shown that there was a need to focus the player’s attention away from the gaming device’s screen during such gameplay situations to avoid dangerous encounters [59]. Aerobics moves can be executed with or without arm movement, indoors as well as outdoors. The combination of indoor and outdoor exercises in an exergame application is a novelty and offers a vast variety of different endurance workout opportunities to meet individual exercise preferences. Instructional videos are available for all exercises (strength, aerobics, balance and flexibility) to ensure correct and safe execution. The algorithms for the individualized exercise training are based on the ACSM and EACPR principles of exercise training [46, 47] and several exercise intervention studies in normal and overweight subjects and patients [60–63]. The training progression in the game is monitored through the repetition of the baseline tests and subsequent adaptions in the exercise/intensity selection. Specific test scores are linked to specific exercises and difficulty levels, so that for example players with higher scores in the STS will get a higher (entry) difficulty level for a specific leg strength exercise than players with a lower score. In addition to these adaptions, the user has the option to rate the intensity of each exercise upon its completion, directly affecting the intensity of the next workout. The rating scale used is a simplified version of the 15-point Borg Scale [64]. In simple terms, a rating of “too hard” will result in a lower difficulty/intensity level in the next workout; a rating of “too easy” will result in a higher difficulty/intensity level in the next workout. Five consecutive ratings of “just right” of the same exercise will result in the suggestion of a higher intensity level in the next workout. The combination of objective as well as subjective measures to control the training progression was chosen to enable unbiased feedback to the player through the repetition of the baseline tests while keeping the player in control of the game at all times, thereby facilitating the long-lasting enjoyment and high levels of self-efficacy that are known to positively influence exercise adherence [49].

**Fig. 3:**
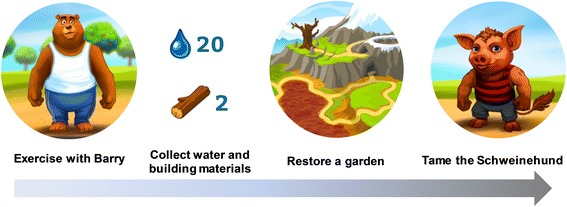
Illustration of the gaming concept of MOBIGAME

**Fig. 4:**
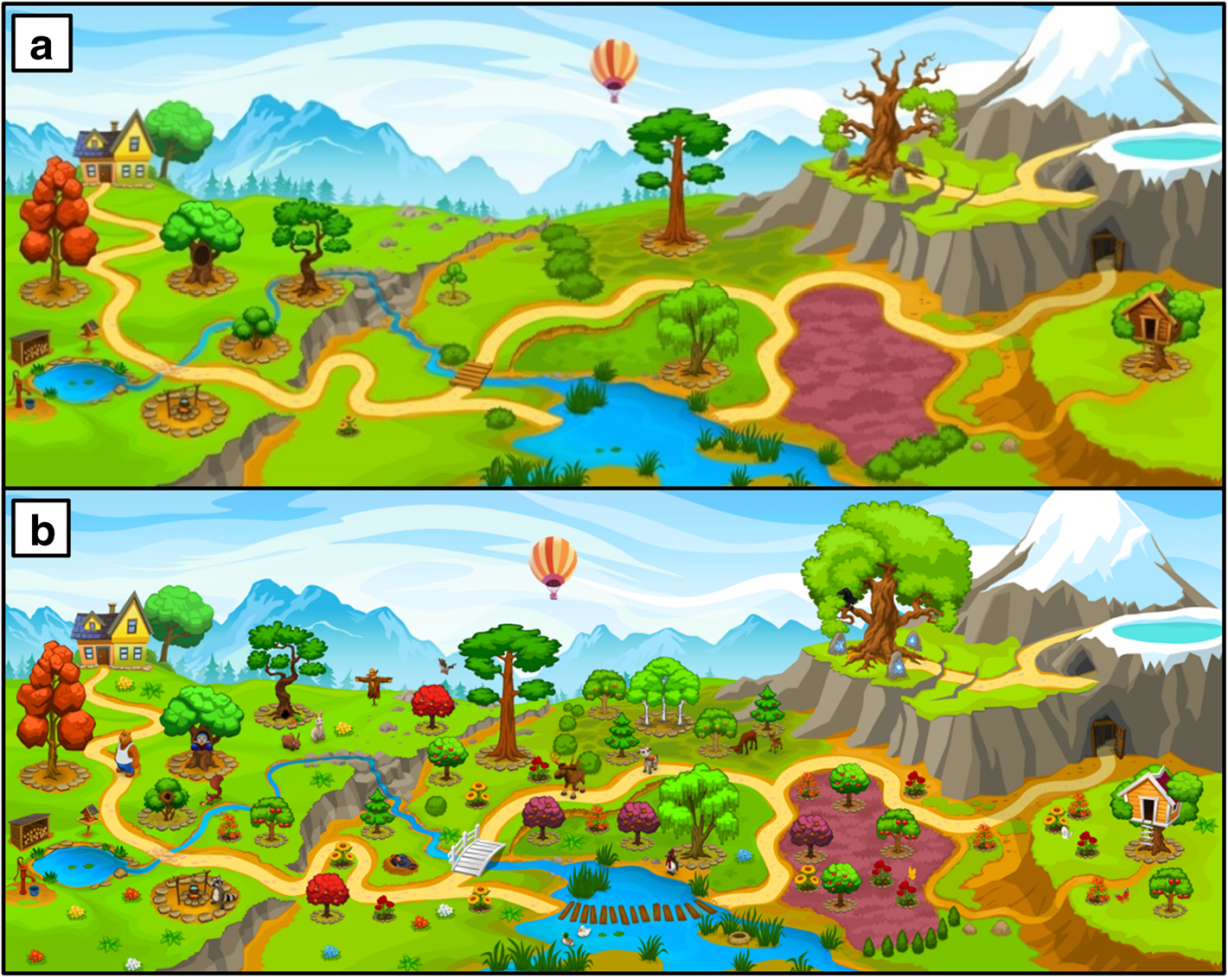
Illustration of the undeveloped garden before starting the exercise (**a**) and the fully developed garden as a result of regular exercise (**b**)

**Fig. 5:**
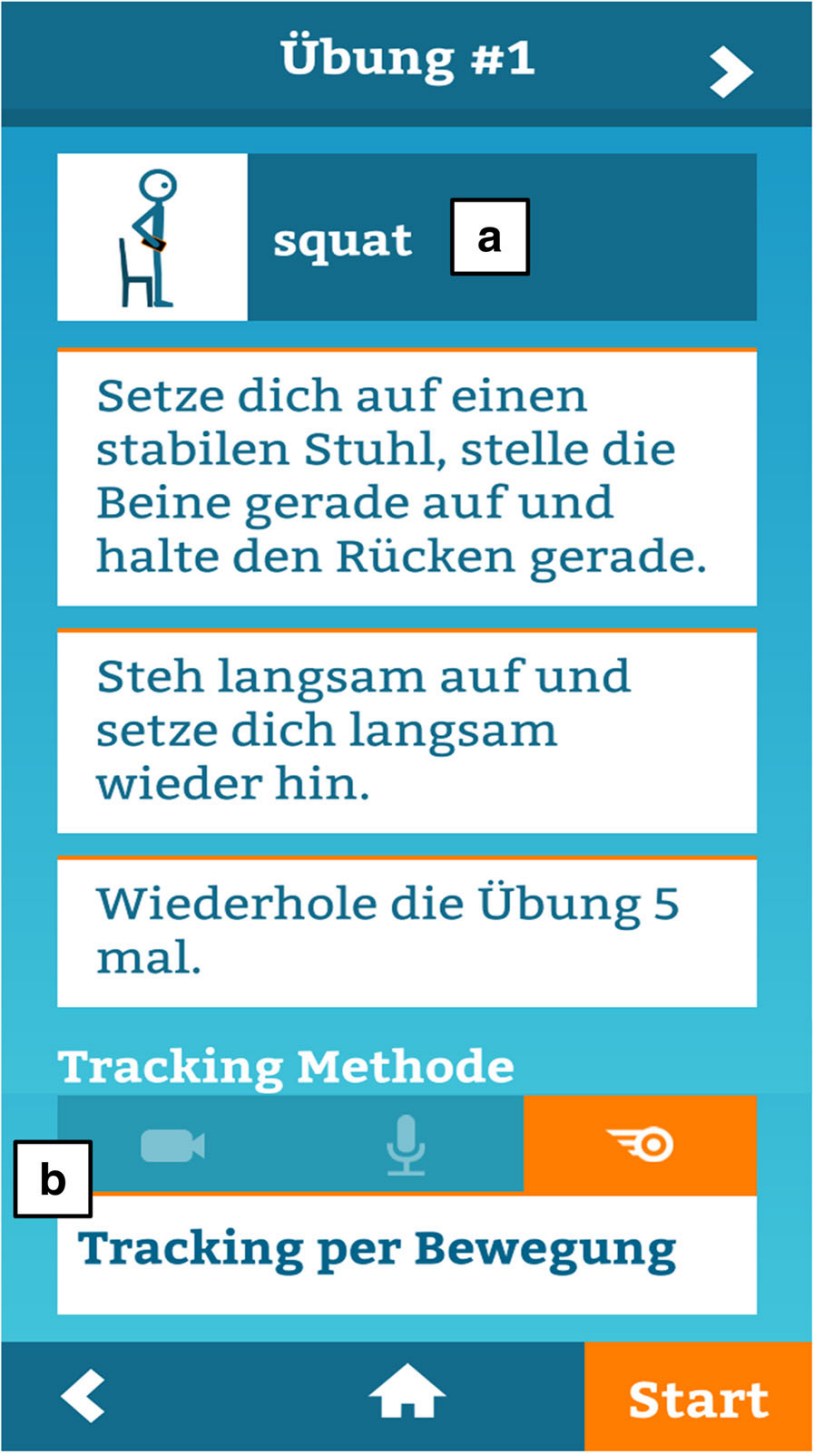
Screenshot of the exercise mode: (**a**) Step-by-step instructions, including a video of an animated stick figure, are available for all exercises to ensure correct execution. (**b**) Tracking of exercise execution via camera, audio sensor or motion sensor tracking

**Fig. 6:**
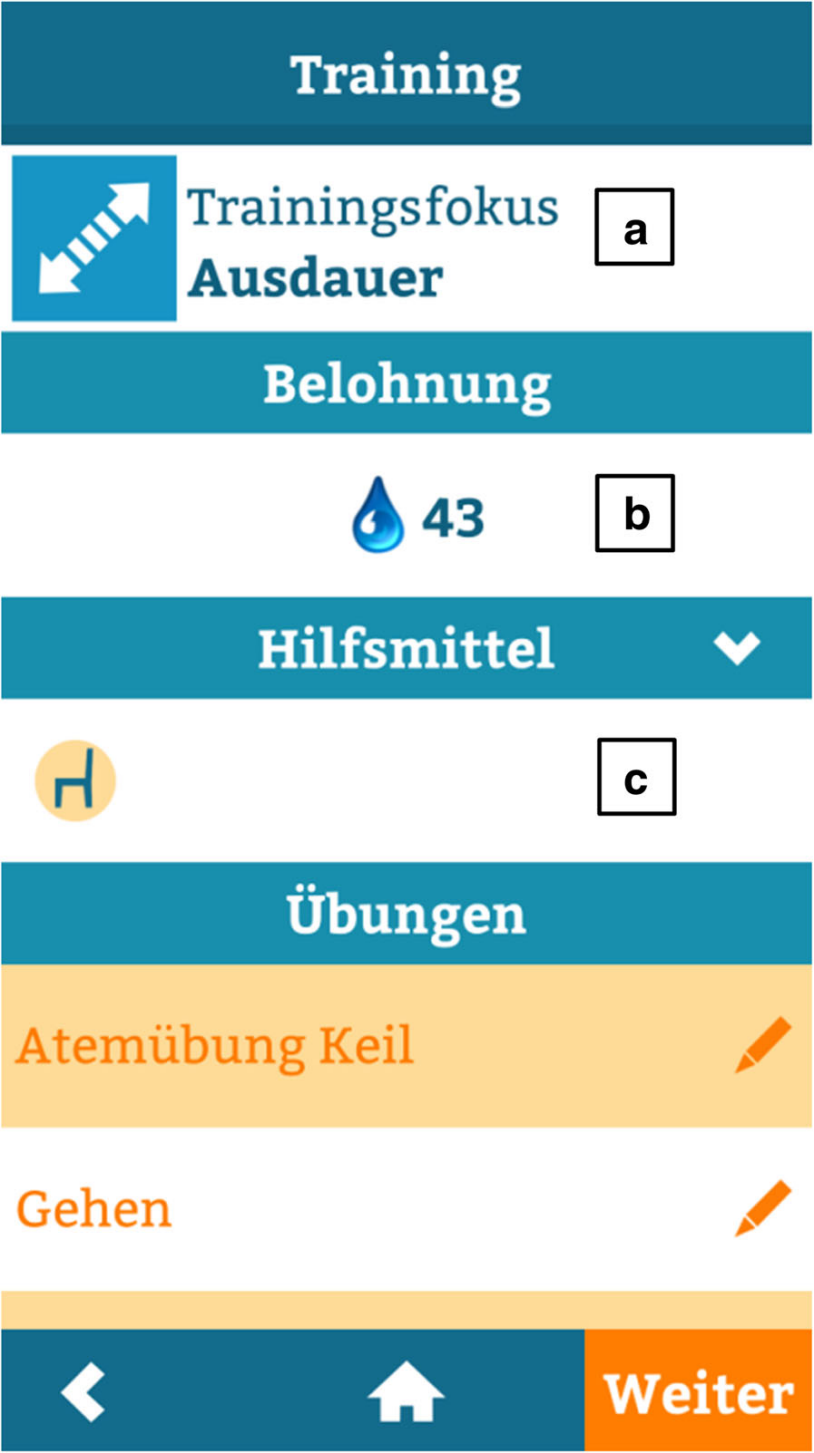
Screenshot of the exercise mode: Training focus (**a**), type of in-app reward (**b**) and required materials (**c**) are clearly stated before the start of the training

**Fig. 7:**
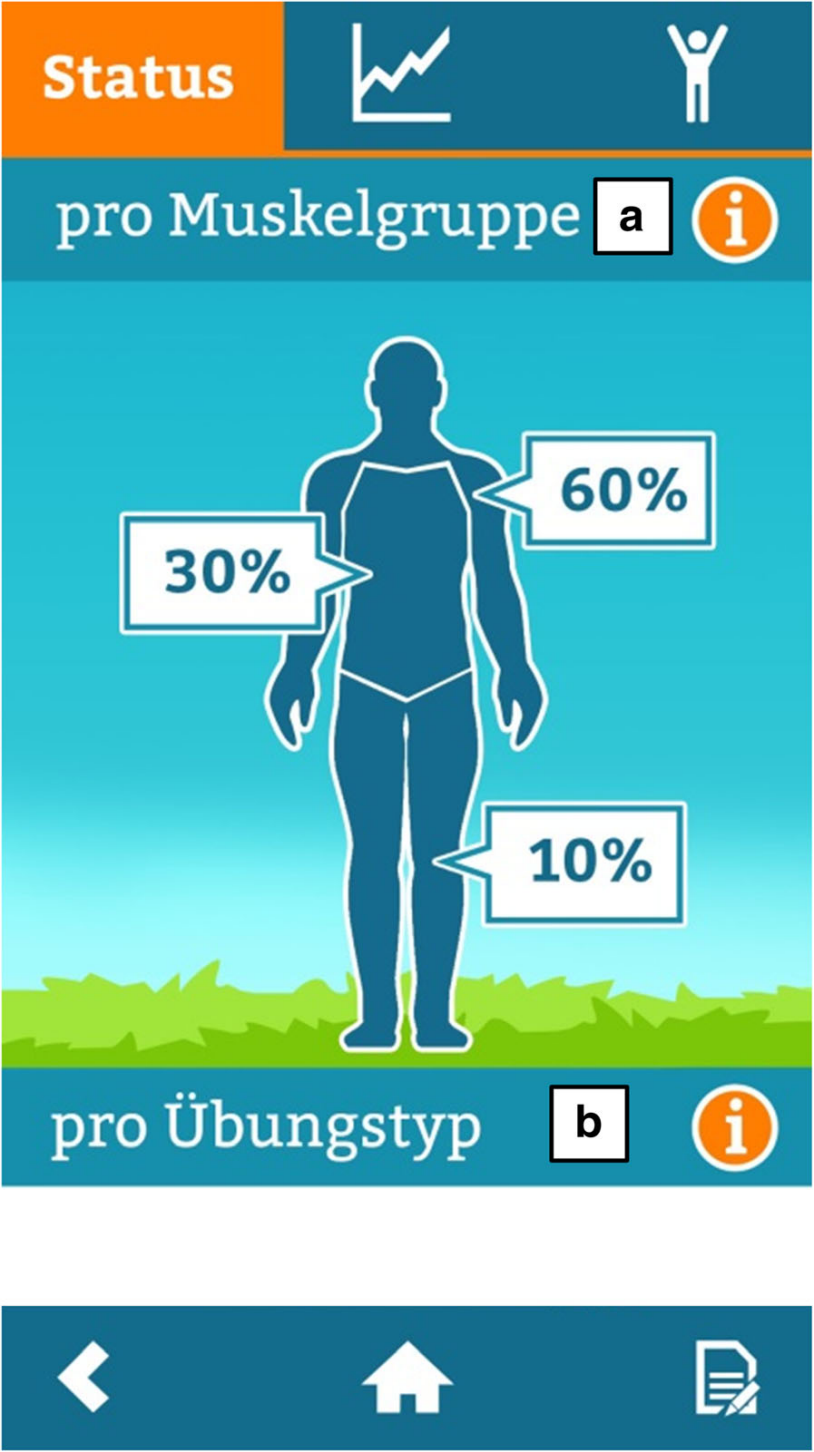
Screenshot: Overview of completed exercises by muscle group (**a**) or by type of exercise (**b**)

**Fig. 8:**
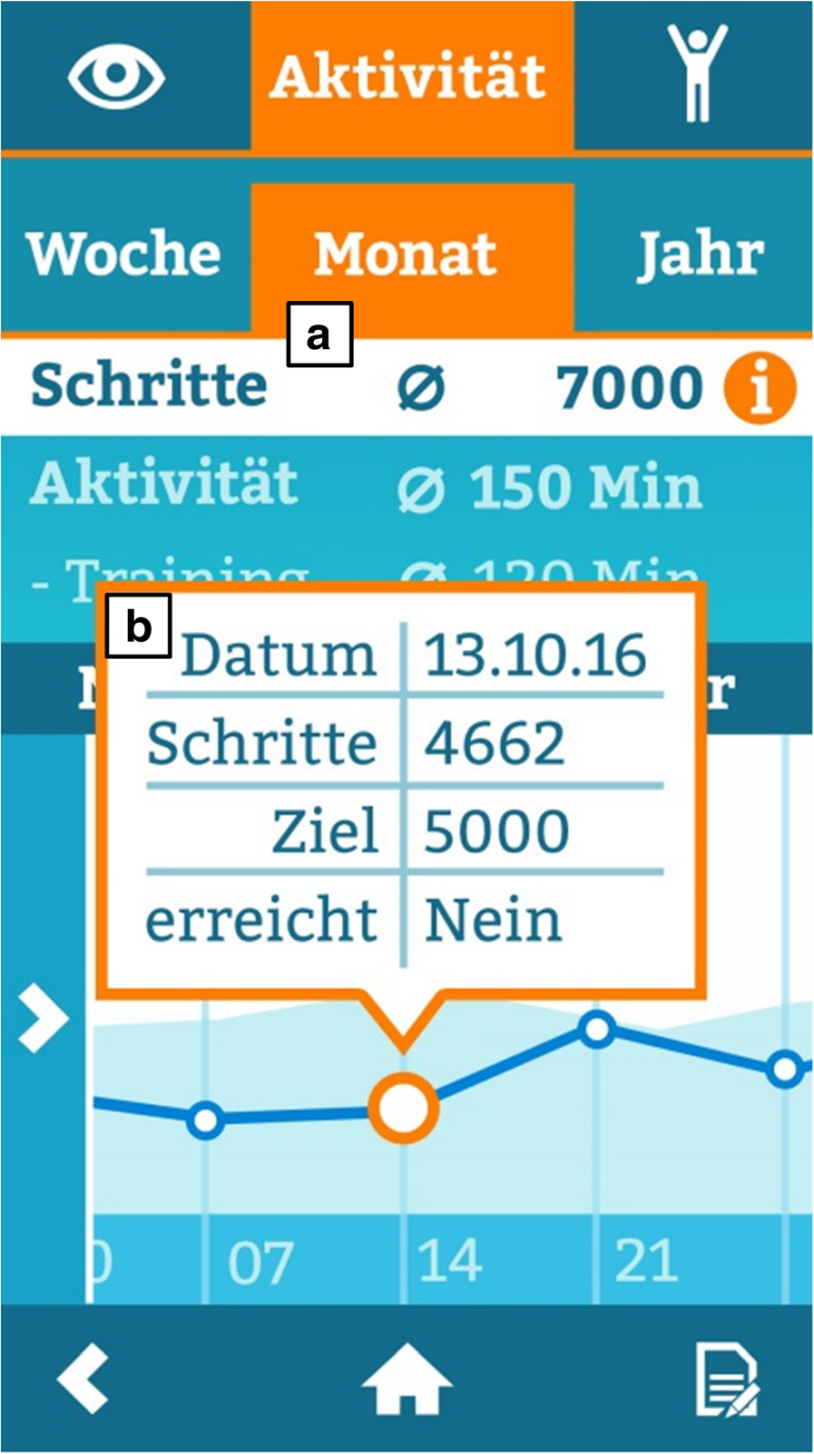
Screenshot of activity overview: (**a**) Average steps and average minutes of activity per week, month (displayed) or year. (**b**) Day view of steps walked, daily step goal and information if step goal was reached

**Fig. 9:**
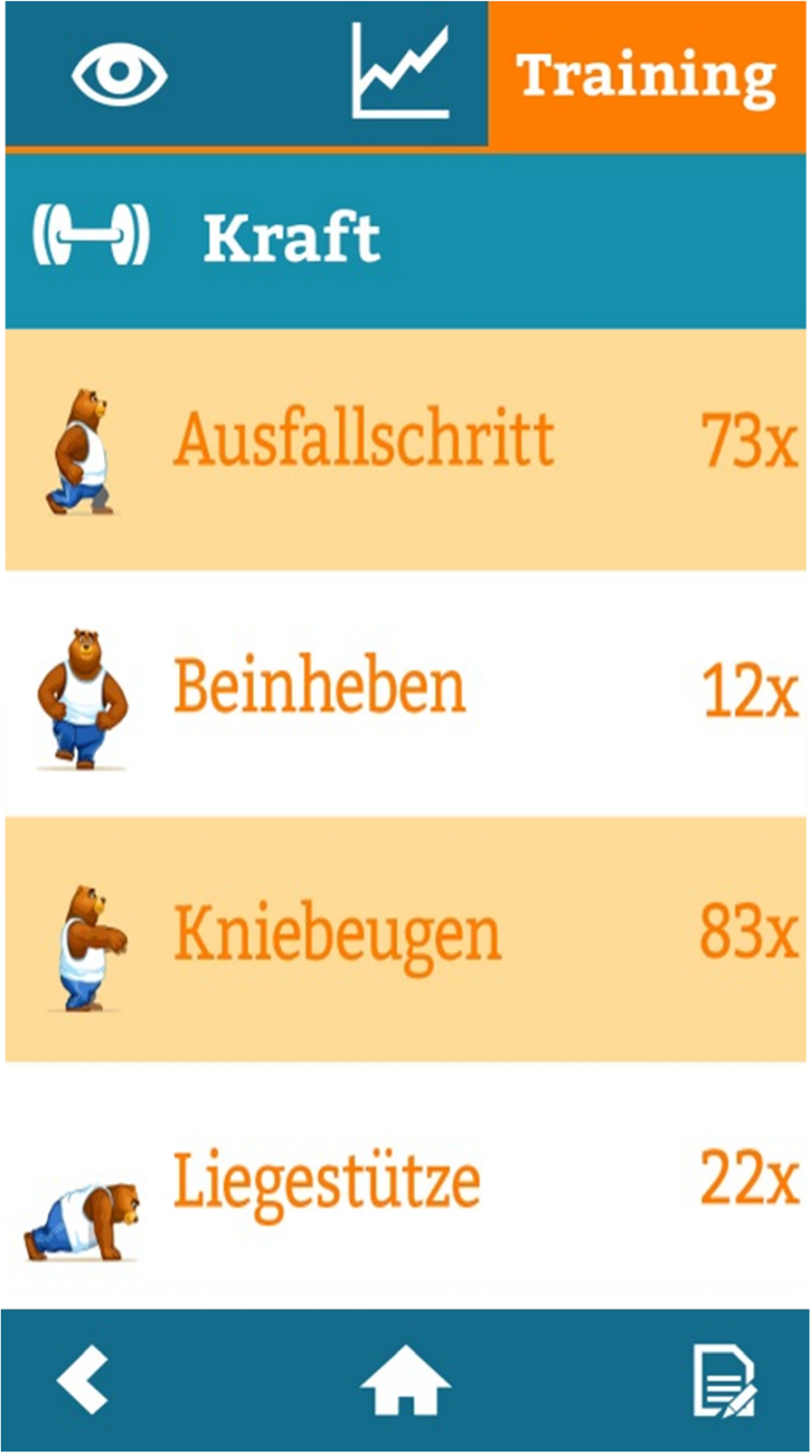
Screenshot of training overview: Total number of completed strength exercises. Exercises displayed are lunge (“Ausfallschritt”), leg lift (“Beinheben”), squat (”Kniebeuge”) and push-up (“Liegestütze”)

A key element of the story line of MOBIGAME is the restoration of a garden. The garden used to be a beautiful place until the Schweinehund came and destroyed it, causing all of the animals that used to live in the garden and help maintain it to leave. In German, “innerer Schweinehund” (inner swine hound) is used as a self-depreciating idiom denoting a form of lazy procrastination, usually of the physical kind, that needs to be overcome to get yourself going. The player’s task is now to help restore the garden by planting trees and flowers and thereby attracting the animals to come back into the garden while at the same time taming the Schweinehund. The garden as the main setting was purposefully chosen as gardens and the activity of gardening have been shown to be highly appealing to the target group [65] and to be effective in reducing stress and stress related-illness such as cardiovascular diseases, depression, reduced immune function and chronic fatigue [66]. In this regard, it has been shown that simply viewing a green space through a window can relax people and reduce stress levels [66, 67]. In our approach, the viewing of this green space (i.e., the garden) occurs through the smartphone screen. Particularly the engagement in gardening activities is very effective in alleviating stress and has been shown to significantly decrease cortisol levels [68]. Research has further shown that nurturing plants from seed to maturity evokes feelings of curiosity, competence and enjoyment, all of which contribute to successful stress management [69, 70]. While we are aware that artificial gardening on a smartphone screen is not identical to an actual gardening experience, the positive and de-stressing character of this activity may still partially be transmitted to the player The animals are humanized (walk upright, wear clothes), always friendly and very likable and represent typical human character traits. Some have even acquired typical human vices such as laziness, sulkiness, contempt or moodiness that the player can help them break by playing the game regularly and thus being regularly physically active. Every activity (in-app workouts as well as steps walked during the day) is rewarded with water or building materials needed to restore the garden. When designing the game, close attention was paid to defined principles and mechanisms such as the inclusion of rules, clear but challenging goals, fantasy, progressive levels of difficulty, interactivity, player control, uncertainty, feedback and a social element [71, 72]. The game mechanics are further anchored in the 40-item CALO-RE taxonomy of behavior change techniques [73]. In this regard particularly “goal setting,” “action planning” and “providing rewards contingent on successful behavior” (i.e., the player sets the goal to complete a super-challenge that requires regular activity on several consecutive days in order to receive a special reward), as well as “providing feedback on performance” (i.e., all of the player’s activity and achieved goals are recorded and summarized in a general overview), are implemented in the game mechanics to help players integrate more PA into their daily routines. “Environmental restructuring” is used quite visibly in the main story line (restoring of the garden) while at the same time and more subtly leading to the development of exercise habits and routines (“prompting practice”) that are supported by the “use of follow-up prompts” (i.e., reminder notifications). The players are encouraged to seek support from the humanized animals to achieve their goals (“elicit social support”) and to keep the Schweinehund away, as failing in doing so will interfere with a successful garden restoration (“fear arousal”). As its ability to provide (lasting) enjoyment is critical to the success of any game, the focus was laid most importantly on this specific factor [74]. While it has been pointed out that for serious games the fun may only need to exceed its traditional analog (health class, lectures from care providers, etc.) to be considered successful [75], we aimed to create an appealing exergame that players want to play because it gives them pleasure by featuring just the right balance between fun and seriousness [51, 76]. Figures 3, 4, 5, 6, 7, 8 and 9 show screenshots of MOBIGAME to illustrate how the gameplay and exercises work from the user perspective.

At the end of the baseline examination, MOBIGAME is installed on the participants’ smartphones (Fig. 2). The participants only receive very basic instructions on how to use and control the app since MOBIGAME was designed to be self-explanatory including a built-in tutorial that explains all of the game’s features to the player at the appropriate time. Participants further do not receive any instructions on how often to use the app because this study seeks to evaluate the motivating character of MOBIGAME by itself and thereby its suitability to increase regular PA.

#### Control intervention details

The control intervention focuses on promoting daily PA through a one-time lifestyle counseling, which will be administered by a sports medical expert. All participants in the control group receive a booklet containing information on the benefits of regular PA for health, opportunities to integrate more PA into the daily routine and illustrations of recommended exercises and activities (comparable to the exercises included in MOBIGAME) as well as 24 weekly exercise logs [77] to check off completed workouts and record additional activities. Increases in daily PA in the control group are possible but are not expected to be of the same extent or persistence as in the experimental group because a daily reminder and the motivating character of a game are missing.

### Study data

#### Characterization of participants

Baseline data of participants are assessed during the recruitment process and within the baseline assessment (see Fig. 2). Demographic data (sex, age, socioeconomic status, smoking status), anthropometric data (weight, height, waist and hip circumference, body composition) and resting blood pressure are assessed by a sports medical expert. Medical history (including drug history and concomitant medication) as well as allergies are assessed during a general physical examination by a physician. PA is assessed using the Freiburg Questionnaire of physical activity [78] as part of the screening during recruitment.

#### Primary outcome

The primary outcome is daily PA measured as steps per day after 24 weeks. It is assessed in a 1-week tracking period during the week before the start of the intervention as well as during the first week after the 24-week intervention using a VivoFit 2 pedometer wristband (Garmin International Inc., Olathe, KS, USA). The displays of the wristbands in both groups will be irreversibly blackened out so that the daily activity can only be viewed by the study personnel via the wristband’s software and a motivating character of PA feedback by the device can be ruled out.

#### Secondary outcome

Participants’ exercise behavior as well as patterns of MOBIGAME use is analyzed in the intervention group via the app’s usage log. The log can be accessed through each participant’s smartphone to draw conclusions regarding the sustainability of player motivation as well as exercise adherence. The usage data will give information about whether goals of daily PA as well as exercise goals are met, how many workouts are completed, how many workouts are done voluntarily and what influence the suggestions of specific workouts as well as the reminder to be physically active have on players’ exercise behavior. The usage data are further used to analyze which workouts are preferably chosen, which are less popular and whether MOBIGAME use can help players reach and maintain 150 min of moderate PA per week.

In the control group, adherence to the exercise regimen, as suggested during the one-time lifestyle counseling, will be self-recorded by the participants in 24 weekly exercise logs (see above).

Further outcomes are described in detail in Additional file 2.

### Timeline

The duration of the entire MOBIGAME project is 50 months (January 2014 to February 2018). Development of MOBIGAME was completed in February 2016. Recruitment of participants started in January 2016 and is expected to end in June 2017 (18 months). Examinations started in August 2016. The last participant is expected to finish the intervention in December 2017. Altogether, the period between “first patient in” and “last patient out” is 20 months.

### Sample size

Determination of sample size was based on the primary outcome. On the basis of a previous study [43] and our own experience, we assumed that the expected difference in daily PA after 24 weeks between participants in the experimental intervention and those in the control group would be 2500 steps per day. We further assumed that the standard deviation given either study group would be 3000 steps per day [43]. By including daily PA (steps per day) at baseline as a covariate in the analysis, we aim to further reduce error variability and therefore assumed that the correlation between baseline and outcome daily PA will be 0.7. With a significance level of 0.05 (2-sided), the sample size needed to attain a targeted power of 90% for showing superiority of the experimental intervention over control was determined as a total of 34 participants (17 in each group). While we aim to achieve complete capture of all data from all participants, it is unavoidable that some participants will fail to provide outcome data. We will therefore increase the proposed sample size by 20%, so that we will include a total of 42 participants (21 in each group).

### Recruitment

Participants are recruited in cooperation with the Clinic of Endocrinology, Diabetes and Metabolism of the University Hospital Basel, Switzerland as well as on the basis of the database of the Diabetesgesellschaft Region Basel, Switzerland. Further, subjects will be recruited through online and newspaper advertising as well as by word of mouth. Patients who seem to be eligible based on their medical records are contacted via email, telephone or personal inquiry. They then receive a questionnaire via email to verify insufficient levels of PA as well as other general eligibility before an appointment for the first day of examination, including the personal assessment of final eligibility, is scheduled at the Department of Sport, Exercise and Health of the University of Basel.

### Assignment of interventions

#### Allocation and blinding

Participants are allocated at random and in equal numbers to one of the two groups. To achieve this, permuted block randomization with randomly varying block sizes is applied. The randomization list was generated in advance using R version 3.2.3 (R Foundation for Statistical Computing, Vienna, Austria) and the R add-on package blockrand version 1.3 [79] and was transmitted using sequentially numbered, opaque, sealed envelopes [80] by a study assistant not involved in the measurement appointments. Another study assistant receives the envelope on the second visit after all measurements are completed and immediately prior to the distribution of MOBIGAME or the one-time lifestyle counseling, respectively. Through the randomly varying block sizes, which were deliberately not disclosed, study assistants involved in both the measurement appointments and the distribution of MOBIGAME or the one-time lifestyle counseling, respectively, as well as the investigator organizing the appointments cannot know which upcoming participant will be allocated to which of the two groups. All other study personnel and all outcome assessors (with the exception of the telephone interviewers) are blinded with respect to group allocation. No unblinding procedures are intended, except for adverse events that require medical care. A blinding of participants regarding group allocation is not possible. However, to minimize performance bias, participants do not receive detailed information about the other intervention [81].

### Data collection

All measurements of this study are performed by standardized procedures and the assessment staff uses standardized instructions for all measurements to ensure equal testing conditions for all participants. For a description of the study instruments, see study data (above).

To promote participant retention and complete outcome data from all participants, including those who discontinue or deviate from intervention protocols, a total incentive of up to 450 CHF per individual as compensation for their time participating in the study will be provided to all participants. The incentive is not awarded at one time, but is delivered as the participant progresses through the study (150 CHF after completion of the baseline assessment and an additional 300 CHF after completion of the final assessment following the 24-week intervention).

### Data management

To ensure anonymous data handling, sensitive patient data have been previously encoded into a string variable. Data are secured by passwords and only accessible by the responsible staff. The obtained data are digitally stored, backed-up and archived at the Department of Sport, Exercise and Health of the University of Basel. All activity and participant usage data collected by MOBIGAME are stored on the phone’s hard drive and encrypted (and thus secured) with a password chosen by the participant. Entering of this password—and thereby the participant’s consent—is required before exporting these data for analysis post intervention.

### Statistical methods

The statistical analysis will conform to the pre-specified statistical analysis plan described below. Following the intention-to-treat analysis strategy, participants will be analyzed in the groups exactly as randomized.

#### Descriptive analysis

A flow chart of the participants’ progress through the trial will be used for reporting [82]. The number of screened T2DM patients who fulfill the eligibility criteria and the number included in the primary, secondary and further analyses as well as the reasons for exclusion from these analyses will be reported. Summary statistics will be provided for baseline and outcome data, as appropriate. Continuous variables will be summarized using the mean and standard deviation for normally distributed data or the median and interquartile range for non-normally distributed data. Frequency counts and percentages will be used for categorical data. Visual inspection of box plots of steps per day and further outcomes will be used to identify possible outliers in each group (experimental vs. control) to be excluded in sensitivity analyses.

#### Main analyses

The main analyses will be performed on the assumption that missing outcome data are missing at random and therefore will be based on complete cases only.

The primary outcome of daily PA (steps per day) after the 24-week intervention and further outcomes will be analyzed by analysis of covariance (ANCOVA) [83]. The results will be presented as differences in outcome (with 95% confidence intervals) between participants in the experimental intervention and those in the control group, adjusted for the corresponding values at baseline. In the case of chance imbalances at baseline, this will remove a possible bias in the estimate of the effect of experimental intervention over control, while, at the same time, yielding a more precise estimate and accordingly more powerful test for the difference between groups.

Normality will be assessed using normal quantile-quantile plots of the residuals, and to assess variance homogeneity, we will use Tukey-Anscombe plots. If the residual plots indicate departure from model assumptions, suitable transformations of the outcome will be considered [84].

For the secondary outcome, each group will be analyzed descriptively with respect to program adherence.

Finally, correlations between MOBIGAME adherence and acceptance of MOBIGAME (TAM questionnaire score, see Additional file 2) will be analyzed using Spearman’s rank correlation coefficient.

#### Sensitivity analyses

The effect that any missing outcome data might have on results will be assessed through sensitivity analyses based on imputed data sets. Dropouts (essentially, participants who are lost to follow-up) will be included using imputation methods that allow for the uncertainty about the imputed values. While a few missing values generally present a minor nuisance, a substantial number of missing values is a major threat to a trial’s integrity [85]. If omitting all patients with incomplete data will result in a large proportion of the data being discarded, we will use multiple imputation where missing data are replaced by a set of plausible imputations generated from the patient’s available data [86]. To achieve this, imputation models will need to be developed based on the majority of patients with complete data. Variables included in these models will be carefully chosen, and the study team will discuss whether this choice makes the underlying assumption that any systematic difference between the missing and the observed outcome data can be explained by differences in observed (baseline) data plausible. Rubin’s rules will be used to combine results across ANCOVA models based on the imputed data sets (at least five imputed data sets will be created) and so give an overall estimate of the effect of experimental intervention over control on the primary and further outcomes.

#### Statistical software

Up-to-date versions of SAS (SAS Institute Inc., Cary, NC) and R (R Foundation for Statistical Computing, Vienna, Austria) will be used for statistical analysis and graphics.

### Quality assurance and monitoring

Written standard operating procedures are used for all measurements to ensure data quality. The state of recruitment, patient participation and consent withdrawals are reported regularly to the project manager. Data completeness and plausibility as well as control of correct randomization/allocation of patients are verified regularly. Any deviation from expected standards are reported to and discussed with the project manager.

### Ethical considerations

The health risks of MOBIGAME use are negligible. To minimize the risk for any cardiorespiratory adverse events, all participants are required to undergo an extensive physical examination including resting and exercise electrocardiography (see Additional file 2) before receiving MOBIGAME as part of the intervention treatment. Overload of soft tissue may be in principle possible but seems very unlikely because of the implemented training structure that begins with very light intensity and duration and is based on the individual results from the baseline testing (6MWT and STS). Training progression is also tailored and can be individually and manually adjusted at any time, thereby further minimizing the risk for adverse events. In addition, at first use of MOBIGAME the player is instructed to contact the primary care physician (or study personnel) should any unexpected adverse events occur during use and to call the emergency number for emergencies.

In contrast, the potential benefits of MOBIGAME use significantly exceed the risk of adverse events and include increased cardiorespiratory fitness and leg strength, improved glucose metabolism and diabetes management as well as lower cardiovascular risk and ultimately an improved quality of life. Accident insurance is provided by the University of Basel for all participants.

## Discussion

PA is a crucial component in the prevention as well as treatment of T2DM [7–12] and its comorbidities [13–15]. Despite the many individually relevant health benefits, adherence to PA-promoting intervention programs in T2DM is generally low and their mid- to long-term effectiveness therefore often limited [27]. On the basis of our extensive review of the current exergaming literature [34] and our own preliminary work examining the intensity of indoor exergaming on cardiorespiratory exertion in T2DM individuals [33], it has been shown that serious exergames as well as mobile app-based programs [38] principally have the potential to be effective treatment options, especially because they seem to at least partly solve the adherence problem [28, 29]. The MOBIGAME application is a milestone in the exergaming approach as it is the first such application seeking to improve physical activity in T2DM individuals by offering individualized and structured strength and endurance workouts as well as balance and flexibility exercises, which are embedded in the game’s story line. All workout intensities are based on the fitness assessment of the player through established field tests that are also part of the story line. MOBIGAME offers the unique opportunity to more sustainably improve adherence to exercise through game enjoyment combined with convenience and individualized goals that give the users immediate feedback and credit for their accomplishments and thereby increase motivation to progress in the game [55]. In contrast to traditional exergames, MOBIGAME offers a large variety of sensor-tracked exercise modes that facilitate workouts indoors as well as outdoors and thus gives the users the opportunity to be physically active wherever they are.

This study will comprehensively evaluate the effectiveness of MOBIGAME as a highly innovative, mobile and individualized home-based treatment option for T2DM patients on sustainably improving daily PA (primary outcome) and several health parameters in the mid to long term. The study aims to assess whether a cutting-edge exergaming application, developed by sports scientists and professional game developers in a close collaboration over the course of 24 months, is superior to traditional home-based patient guidance. If so, this application may cover a gap in the treatment of those patients with T2DM not willing or not able to participate in structured group programs and could at the same time reduce time-consuming and personal-intensive face-to-face as well as telephone consultations (or at best make them redundant). Last but not least, this study will add considerably to the understanding of whether a mobile phone-based game application is an option to sufficiently address the problem of program adherence in PA-promoting interventions and provide relevant information for the general transferability of this application for use in other chronic diseases.

### Trial status

At the time of manuscript submission (Version 1, 27 October 2016), examinations have started (see timeline).

## Additional files

Additional file 1: Standard Protocol Items: Recommendations for Interventional Trials (SPIRIT) 2013 checklist. (DOC 121 kb)
Additional file 2: Study data – further outcomes. (DOCX 49 kb)

## Abbreviations

6MWT: 6-Minute Walk Test
ACSM: American College of Sports Medicine
ANCOVA: analysis of covariance
BMI: body mass index
EACPR: European Association for Cardiovascular Prevention & Rehabilitation
EKNZ: Ethics Committee Northwest/Central Switzerland
FACIT: Functional Assessment of Chronic Illness Therapy
FOPH: Federal Office of Public Health
HbA_1c_: glycated hemoglobin
HDL: high-density lipoprotein
HOMA: Homeostasis Model Assessment
HR_max_: maximum heart rate
HRQOL: health-related quality of life
IMI: Intrinsic Motivation Inventory
LDL: low-density lipoprotein
MET: metabolic equivalent
PA: physical activity
PRS: Protocol Registration and Results System
RPE: ratings of perceived exertion
RVA: retinal vessel analyzer
SF-36: 36-Item Short Form Health Survey
SNSF: Swiss National Science Foundation
STS: Sit-to-Stand Test
T2DM: type 2 diabetes mellitus
TAM: Technology Acceptance Model
VO_2max_: maximum oxygen consumption
VO_2mean_: mean oxygen consumption
VO_2peak_: peak oxygen consumption
