# Supplementary material for: Mobile Exergaming for Health—Effects of a serious game application for smartphones on physical activity and exercise adherence in type 2 diabetes mellitus—study protocol for a randomized controlled trial
Source: Trials. 2017 Mar 6;18:103. doi: 10.1186/s13063-017-1853-3 (PMC5339965; doi:10.1186/s13063-017-1853-3)
Supplement: Additional file 2: — Study data – further outcomes. (DOCX 49 kb) [file 13063_2017_1853_MOESM2_ESM.docx]

# Electronic supplementary material

## Study data – further outcomes

The following parameters are measured at baseline as well as after the 24-week intervention:

Self-determination
Consisting of intrinsic motivation, perceived competence, perceived choice, and perceived usefulness, self-determination is measured via the Intrinsic Motivation Inventory (IMI) score. The IMI is a multidimensional Likert-type rating scale that assesses the central motivational structures underlying self-determination [1]. It has been shown to possess good internal consistency (Cronbach’s alpha = 0.92) and test-retest reliability (intraclass correlation coefficient = 0.77) in other populations [1] and has been used in previous virtual reality PA studies [2–6].

Aerobic capacity
Aerobic capacity is measured as VO_2peak_ during an all-out bike spiroergometry using the mobile METALYZER 3B spirometry system (Cortex Biophysik GmbH, Leipzig, Germany). After a 3-minute warm-up phase at 25 W, workload increases by 15 W/min until subjects’ subjective exhaustion. Pedaling frequency has to be above 60 rpm throughout the entire test. Breath by breath gas analyses is measured permanently, ratings of perceived exertion (RPE) [7] and blood pressure are measured at rest, after warm-up and every two minutes until exhaustion. The blood lactate concentration is measured at rest and at maximum performance as well as 1, 3 and 5 minutes after the end of the exercise test. Throughout the entire duration of the test and until exertion, cardiac function is monitored with a 12-channel electrocardiogram.

Maximal 6-minute walking distance
Maximal 6-minute walking distance is assessed with the 6-minute walk test, an established measure of functional exercise capacity commonly assessed in T2DM [8].

Leg strength endurance
Leg strength endurance is assessed as the maximum number of repetitions in the Sit-to-Stand Test [9].

Maximal isometric force
Maximal isometric force and rate of force development are measured in a double-limb leg press on an isokinetic dynamometer (D&R Ferstl, IsoMed 2000, Hemau, Germany) [10]. During the measurement the seat back is reclined to 50°. Hip and knee angles are individually adjusted at 90° and 100°, respectively, while the hip is additionally fixed with a belt. The testing condition will consist of two maximal contractions of five seconds with a recovery time of 30 seconds in between two contractions [10]. As decreases of skeletal muscle mass and muscle quality especially in the lower limb are common in older adults with T2DM [11] and often contribute to fatigue [12] and a reduced HRQOL [13] the assessment of leg strength is an important outcome of the MOBIGAME intervention.

Glucose metabolism
Glucose metabolism (fasting glucose, glycated hemoglobin (HbA_1c_), fasting C-peptide, fasting insulin levels) measured by standard laboratory analysis of venous blood. The Homeostasis Model Assessment (HOMA) index is used to quantify insulin resistance and ß-cell function by implicating the measured fasting insulin and fasting glucose concentrations.

Inflammatory markers
Inflammatory markers such as total cholesterol, low-density lipoprotein (LDL)- and high-density lipoprotein (HDL)-cholesterol, triglycerides, apolipoprotein B, irisin, adiponectin and interleukin-6 levels, measured by standard laboratory analysis of venous blood.

Macrovascular function
Macrovascular function (peripheral and central blood pressure, pulse wave reflection as augmentation index and arterial stiffness as aortic pulse wave velocity) is assessed using a cuff-based oscillometric measurement device (Mobil-O-Graph, I.E.M GmbH, Stolberg, Germany) by the application of the ARCSolver algorithm to pulse wave signals acquired with the Mobil-O-Graph device [14]. Peripheral and central blood pressure, pulse wave reflection as augmentation index and arterial stiffness are commonly used as independent predictors to assess the cardiovascular risk [15]. These methods are valid and reliable ways to assess arterial stiffness, central blood pressure and pulse wave reflection in a Caucasian population [14,16].

Microvascular function
Microvascular function (retinal vessel diameters) is analyzed using the Retinal Vessel Analyzer (RVA, IMEDOS Systems, Jena, Germany). Three valid images from the retina of the left and the right eye with an angle of 30° and with the optic disc in the center are taken per visit in order to analyze retinal vessel diameters as previously described [17] and applied in a previous study [18] using a special analyzing software (Vesselmap 2, Visualis, Imedos Systems). Reliability of retinal vessel diameter analysis has been shown to be high, with inter-observer and intra-observer intraclass correlation coefficients for arteriolar and venular diameter measurements ranging from 0.78 to 0.99 [17,19]. Retinal vessel diameter analysis has been used as a microvascular biomarker and independent predictor of cardiovascular risk and mortality [20–22].

Health-related quality of life
Health-related quality of life (HRQOL) is assessed via the 36-Item Short Form Health Survey (SF-36). This patient-reported survey features a set of generic, coherent, and easily administered quality-of-life parameters, while its vitality scale specifically addresses fatigue measures [23].

Fatigue
Fatigue is measured by use of the Functional Assessment of Chronic Illness Therapy (FACIT) Fatigue Scale. It has demonstrated reliability and sensitivity to change in patients with a variety of chronic health conditions with a high test-retest reliability (intraclass correlation coefficient = 0.95) and a high internal validity (Cronbach’s alpha = 0.96) [24].

Acceptance of intervention
To evaluate the participants’ perceived acceptance of the MOBIGAME intervention, and to find possible correlations with adherence levels, an abridged version [25] of the technology acceptance model (TAM) questionnaire is used in addition in the intervention group post intervention [26].

References

1. Choi J, Mogami T, Medalia A. Intrinsic Motivation Inventory: An Adapted Measure for Schizophrenia Research. Schizophr. Bull. 2010;36:966–76.

2. Lyons EJ, Tate DF, Ward DS, Ribisl KM, Bowling JM, Kalyanaraman S. Do motion controllers make action video games less dedentary? A randomized experiment. J Obes. 2012;2012:e852147.

3. Ijsselsteijn WA, de Kort YAW, Westerink J, de Jager M, Bonants R. Virtual Fitness: Stimulating Exercise Behavior Through Media Technology. Presence Teleoper Virtual Env. 2006;15:688–698.

4. Lyons EJ, Tate DF, Komoski SE, Carr PM, Ward DS. Novel approaches to obesity prevention: effects of game enjoyment and game type on energy expenditure in active video games. J Diabetes Sci Technol. 2012;6:839–48.

5. Lyons EJ, Tate DF, Ward DS, Bowling JM, Ribisl KM, Kalyararaman S. Energy expenditure and enjoyment during video game play: differences by game type. Med Sci Sports Exerc. 2011;43:1987–93.

6. Lyons EJ, Tate DF, Ward DS, Ribisl KM, Bowling JM, Kalyanaraman S. Engagement, enjoyment, and energy expenditure during active video game play. Health Psychol. 2014;33:174–81.

7. Borg GA. Psychophysical bases of perceived exertion. Med Sci Sports Exerc. 1982;14:377–81.

8. Latiri I, Elbey R, Hcini K, Zaoui A, Charfeddine B, Maarouf MR, et al. Six-minute walk test in non-insulin-dependent diabetes mellitus patients living in Northwest Africa. Diabetes Metab. Syndr. Obes. Targets Ther. 2012;5:227–45.

9. Strassmann A, Steurer-Stey C, Lana KD, Zoller M, Turk AJ, Suter P, et al. Population-based reference values for the 1-min sit-to-stand test. Int. J. Public Health. 2013;58:949–53.

10. Donath L, Faude O, Roth R, Zahner L. Effects of stair-climbing on balance, gait, strength, resting heart rate, and submaximal endurance in healthy seniors. Scand. J. Med. Sci. Sports. 2014;24:e93–101.

11. Park SW, Goodpaster BH, Lee JS, Kuller LH, Boudreau R, de Rekeneire N, et al. Excessive loss of skeletal muscle mass in older adults with type 2 diabetes. Diabetes Care. 2009;32:1993–7.

12. Fritschi C, Quinn L. Fatigue in Patients with Diabetes: A Review. J. Psychosom. Res. 2010;69:33–41.

13. Stover JC, Skelly AH, Holditch-Davis D, Dunn PF. Perceptions of health and their relationship to symptoms in African American women with type 2 diabetes. Appl. Nurs. Res. ANR. 2001;14:72–80.

14. Nunan D, Fleming S, Hametner B, Wassertheurer S. Performance of pulse wave velocity measured using a brachial cuff in a community setting. Blood Press. Monit. 2014;19:315–9.

15. Vlachopoulos C, Xaplanteris P, Aboyans V, Brodmann M, Cífková R, Cosentino F, et al. The role of vascular biomarkers for primary and secondary prevention. A position paper from the European Society of Cardiology Working Group on peripheral circulation: Endorsed by the Association for Research into Arterial Structure and Physiology (ARTERY) Society. Atherosclerosis. 2015;241:507–32.

16. Nunan D, Wassertheurer S, Lasserson D, Hametner B, Fleming S, Ward A, et al. Assessment of central haemomodynamics from a brachial cuff in a community setting. BMC Cardiovasc. Disord. 2012;12:48.

17. Hubbard LD, Brothers RJ, King WN, Clegg LX, Klein R, Cooper LS, et al. Methods for evaluation of retinal microvascular abnormalities associated with hypertension/sclerosis in the Atherosclerosis Risk in Communities Study. Ophthalmology. 1999;106:2269–80.

18. Hanssen H, Nickel T, Drexel V, Hertel G, Emslander I, Sisic Z, et al. Exercise-induced alterations of retinal vessel diameters and cardiovascular risk reduction in obesity. Atherosclerosis. 2011;216:433–9.

19. Wong TY, Knudtson MD, Klein R, Klein BEK, Meuer SM, Hubbard LD. Computer-assisted measurement of retinal vessel diameters in the Beaver Dam Eye Study: methodology, correlation between eyes, and effect of refractive errors. Ophthalmology. 2004;111:1183–90.

20. Wong TY, Klein R, Sharrett AR, Duncan BB, Couper DJ, Tielsch JM, et al. Retinal arteriolar narrowing and risk of coronary heart disease in men and women. The Atherosclerosis Risk in Communities Study. JAMA. 2002;287:1153–9.

21. Ikram MK, de Jong FJ, Bos MJ, Vingerling JR, Hofman A, Koudstaal PJ, et al. Retinal vessel diameters and risk of stroke: the Rotterdam Study. Neurology. 2006;66:1339–43.

22. Wang JJ, Liew G, Klein R, Rochtchina E, Knudtson MD, Klein BEK, et al. Retinal vessel diameter and cardiovascular mortality: pooled data analysis from two older populations. Eur. Heart J. 2007;28:1984–92.

23. Ware JE, Kosinski M, Dewey JE. How to score version 2 of the SF-36 health survey: standars & acute forms. QualityMetric; 2001.

24. Chandran V, Bhella S, Schentag C, Gladman DD. Functional Assessment of Chronic Illness Therapy‐Fatigue Scale is valid in patients with psoriatic arthritis. Ann. Rheum. Dis. 2007;66:936–9.

25. Abu-Dalbouh HM. A questionnaire approach based on the technology acceptance model for mobile tracking in patient progress applications. J. Comput. Sci. 2013;9:763–70.

26. Wüest S, Borghese NA, Pirovano M, Mainetti R, van de Langenberg R, de Bruin ED. Usability and Effects of an Exergame-Based Balance Training Program. Games Health J. 2014;3:106–14.
